# A randomized control trial of the effect of yoga on *Gunas* (personality) and Self esteem in normal healthy volunteers

**DOI:** 10.4103/0973-6131.43287

**Published:** 2009

**Authors:** Sudheer Deshpande, H R Nagendra, Raghuram Nagarathna

**Affiliations:** Department of Yoga Research, Swami Vivekananda Yoga Anusandhana Samsthana, Bangalore, India

**Keywords:** Guna, self esteem, Yoga

## Abstract

**Background/Aims:**

To study the efficacy of yoga on *Gunas* (personality) and self esteem in normal adults through a randomized comparative study.

**Materials and Methods:**

Of the 1228 persons who attended motivational lectures, 226 subjects aged 18–71 years, of both sexes, who satisfied the inclusion and exclusion criteria, and who consented to participate in the study were randomly allocated into two groups. The Yoga (Y) group practised an integrated yoga module that included *asanas, pranayama*, meditation, notional correction, and devotional sessions. The comparison group practised mild to moderate physical exercises (PE). Both groups had supervised practices for one hour daily, six days a week, for eight weeks.

*Guna* (personality) was assessed before and after eight weeks using the self-administered “The ’Gita” Inventory of Personality” (GIN) to assess *Sattva, Rajas*, and *Tamas*. Self esteem in terms of competency (COM), global self esteem (GSE), moral and self esteem (MSE), social esteem (SET), family self esteem (FSE), body and physical appearance (BPA), and the lie scale (LIS) were assessed using the self esteem questionnaire (SEQ).

**Results:**

The baseline scores for all domains for both the groups did not differ significantly (*P* > 0.05 independent samples t-test). There were significant pre-post improvements in all domains in both groups (*P* < 0.001 paired t-test). The number of persons who showed improvement in *Sattva* and decrease in *Tamas* was significant in the Y but not in the PE group (McNemar test). The effect size for self esteem in the Y group is greater than for the PE group in three out of seven domains.

**Conclusions:**

This randomized controlled study has shown the influence of Yoga on *Gunas* and self esteem in comparison to physical exercise.

## INTRODUCTION

The World Health Organization defines “health” as a dynamic state of complete physical, mental, social, and spiritual wellbeing and not merely as the absence of disease or infirmity.[1] A century ago, most medical research and expertise was directed towards the handling of problems such as nutritional deficiencies and epidemics. With the advances in medicine over the years, the types of ailments being treated have changed. Today, most of the common health problems are traceable to lifestyle. Despite advances in pharmacotherapeutics, the need for lifestyle modification, including physical exercise, healthy habits (abstention from smoking), proper diet, and stress adaptability have become the cornerstone in the management of most of these chronic ailments.[2] Most systems of complementary and alternative medical modalities (CAM) have become popular because they insist on a healthy lifestyle as a prerequisite to any medication. Thus, the promotion of a positive Quality of Life (QoL) and the movement towards healthier mental, social, and spiritual health has become a necessity which can be improved by community-based, mind-body training programs.[3] The problems of lifestyle (diet, addictive habits, poor physical activity, and poor stress adaptability) are traceable to poor mastery over the mind. Yoga, as a mindfulness practice, tries to correct the basic limitations of the mind by improving self awareness, self control, and self esteem.[4] Due to its strong scriptural and experimental knowledge base, yoga has the potential to become an essential baseline modality in all health work, be it for cure or prevention or the promotion of positive QoL.[5] Yoga offers society a conscious process to solve the problems of unhappiness, restlessness, emotional upsurges, hyperactivity etc.[6] Several studies have revealed that yoga has shown markedly higher scores in life satisfaction and lower scores in excitability, aggressiveness, openness, emotional ability, and somatic complaints. Significant differences were also observed with respect to coping with stress and mood.[7] There are other studies that have independently shown the beneficial effects of yoga on different aspects of health, including physical stamina,[8] visual perception,[9] performance,[10] memory,[11] intelligence,[12] and concentration,[13] using many of the well known standard measures available as research tools. As a holistic science, yoga also provides us with a holistic tool of measurement of the growth of a personality from an instinctual level to higher states of freedom, bliss, and mastery.

The Gita inventory (GIN)[14] is now available as a standardized, psychological tool, and is based on the concept that there are three different levels of human existence in which the mind is always in a dynamic equilibrium between three types of response patterns called *Gunas*. The three patterns are *Sattva* (gentle and controlled), *Rajas* (violent and uncontrolled) and *Tamas* (dull and uncontrolled). In an ideal situation of perfect health, an individual has the complete freedom to use any of these three patterns of responses. Ill health or limited health occurs if *Rajas* or *Tamas* becomes dominant, as one loses freedom and gets habituated to either of these response patterns. Hence, the degree of positive health can be measured by a tool that can grade these three patterns of behaviour.[15]

Yoga concepts also include another important measure of an evolving personality, which is the knowledge about one’s unlimited potential to move towards perfect harmony with Nature. This is reflected to some extent in modern psychology as self esteem with clear-cut definitions and tools for assessment· Hence, this study includes one of the standard available measures called a self esteem questionnaire (SEQ)[16] to see if these concepts correlate with the Gita concepts measured in GIN.

Whereas yoga is getting popular, the relative roles of yoga and physical exercises have not been studied with regard to their effects on *Gunas* and self esteem. Hence, the present study was designed to find out the effect of the Integrated Approach of Yoga Module on *Gunas* and self esteem in normal healthy adults through a randomized controlled trial.

## MATERIALS AND METHODS

Two hundred twenty-six subjects who consented to participate in the study, were randomly allocated into two groups of equal size. Inclusion criteria included (a) normal healthy volunteers, (b) both sexes, (c) age between 18–71 years, and (d) literates. Exclusion criteria: (a) subjects with any ailment, (b) substance abuse, and (c) smokers.

Informed consent was obtained from all the subjects who participated in the study and also from the institutional heads where the classes were conducted. The institutional ethical committee of the parent institution had cleared the project proposal.

This study had a prospective, randomized, single-blind control design to compare the efficacy of yoga (Y) with that of physical exercise (PE) as a control intervention in normal healthy volunteers in a south Indian population. Motivational lectures were arranged in public centers such as colleges, health clubs, Rotary clubs, Lions clubs, and apartment complexes. The classes were planned in five different centers in Bangalore City.

### Randomization

Those who consented to participate in the study and who satisfied the inclusion and exclusion criteria were randomly allotted into two groups by using five different random number tables (different table for each center) generated from the random number generator program.[17] After reading the instructions in the informed consent form about the design of the study, these 226 subjects agreed to be in the allotted group. The experimental group was given Y practices and the control group was given PE for one hour daily on an empty stomach (6 to 7 a.m.). The classes were conducted on six days a week for eight weeks and attendance was maintained by the teachers. Trained experts in either Y or PE conducted parallel sessions for the two groups in different rooms of the same building. It was ensured that there was no interaction between the subjects. The tests were self-administered by encouraging subjects to sit in a quiet place free from distractions and influences from other people, before the intervention and eight weeks after the intervention.

Masking: The answered questionnaires were coded and kept away for future scoring. A psychologist who was not involved in subject allocation or supervision of the classes, scored the questionnaires that were decoded only after the scoring of both pre- and post- data was completed.

### Assessments

Assessments were done using the following questionnaires:

1. Gita Inventory of Personality (GIN): The GIN inventory is based on the concept of *Gunas* (personality) from the Bhagavadgita, a traditional text of yoga, which was developed by Das in 1991. This measure of the three *Gunas* contains ten questions that have three response choices. This test has a test-retest of 0.60 with a confidence level of 99% and has been validated. This is a valid tool for identifying the type of personality.[14]
2. Self-Esteem Questionnaire (SEQ): This multidimensional questionnaire was developed and standardized by Karunanidhi.[15]

### Interventions

#### Yoga group

Table 1 shows the list of practices used for the two groups. The integrated yoga module was selected from the integrated set of yoga practices used in earlier studies on yoga for positive health.[18] This integrated approach is developed based on ancient Yoga texts,[19] to bring about a total development at physical, mental, emotional, social, and spiritual levels.[20] The techniques include physical practices (*Kriyas, asanas*, healthy yoga diet), breathing practices with body movements, and *Pranayama*, meditation, devotional sessions, lectures on yoga, stress management, and lifestyle change through notional corrections for blissful awareness under all circumstances (action in relaxation). Yoga was taught by qualified yoga teachers.

**Table 1:** Details of Yoga and PE practices

Yoga practices		Physical exercise practice	
Duration	Names	Duration	Names
5 minutes	Breathing practices	10 minutes	Warm up exercises
	Hands in and out breathing		(a) Loosening of ankles
	Dog breathing		(b) Knee caps,
	Tiger breathing		(c) Waist,
	Straight leg raise breathing		(d) Spine
			(e) Twisting,
5 minutes	Loosening exercises		(f) Shoulder movements,
	Jogging		(g) Hands movement,
	Forward and backward bending		(i) Wrist movements and rotations
	Side bending		(j) Neck movement and rotations
	Twisting		(k) Head movement and rotations
	Pavanamuktäsana kriya		
		5 minutes	Stretches
25 minutes	*Äsanas*		(a) Leg stretch,
	Standing		(b) Hand stretch,
	Ardha chakrasana		(c) Leg to hand,
	Pada hastasana		(d) Sideward leg stretch (full),
	Privritta trikonasana		(e) Folded leg lumber stretch,
	Sitting		(f) Dog stretch,
	Vajrasana		(g) Tiger stretch, dorsal stretch
	Supta vajrasana		
	Chakrasana		
	*Hamsasana or mayurasana*	10 minutes	Sit-ups (50 to 100 times),
	Prone postures		Push-ups (20 times),
	Dhanurasana		Squats
	Supine postures		
	Sarvangasana	10 minutes	Supine
	Matsyasana		(a) Single leg raising,
	Ardha shirshasana or shirshasana		(b) Alternative leg raising,
			(c) Both leg raising (50 times)
5 minutes	Deep relaxation technique		(d) Coming up and touching the knees to ad and going to forehead and going back..
10 minutes	Pranayama		(e) Cycling
	Kapalabhati		
	Vibhagiya pranayama	10 minutes	Supine rest (Guided relaxation)
	Nadishuddhi pranayama		
	Sitali, sitkari and sadanta	10 minutes	Dynamics
	Bhramari pranayama		(a) Forward Backward bending,
	Nada anusandhana		(b) Side bending,
	Or		(c) Bending and twisting
			(Simple and legs apart),
	Meditation: Om meditation		(d) Twisting
10 minutes	Bhajans/Lectures	5 minutes	Lectures

### Physical exercise group

The set of physical exercises chosen for this study were the standard practices[21] to provide mild to moderate exercises designed by experts in physical education and were taught by trained physical education teachers. This group also had interactive lectures on healthy lifestyles, including diet habits and stress management based on modern medical knowledge. The daily sessions began with short five-minute talks on lifestyle and health covering the topics of (a) healthy diet (six talks) such as classification of foods, energy-yielding foods, the role of animal fat and relationship to cholesterol, vegetarian *v*s nonvegetarian diet, value of fiber etc., (b) value of exercise and health (six sessions) explaining different type of exercises, its effect on muscles, effect on joints, value of regular sport activity etc, (c) bad effects of smoking (four talks), alcohol, and other chemical abuse (two sessions), (d) effect of mental stress on health and the role of physical exercise in the management of stress. This was followed by the practice of the physical exercises for 45 minutes with enough rest in between. The sessions ended with ten minutes of relaxation (without guided instructions) in a supine position.

### Data extraction

The scoring of the questionnaires was carried out as per the instructions given in the manuals. The structures of these questionnaires are described below:

GIN: This inventory has ten questions to evaluate *Tamas, Rajas, and Sattva Gunas*. The score value of weightage of an item indicating *Sattva* is 3, for an item indicating *Rajas* is 2, and for an item indicating *Tamas* is 1. It classifies people as being predominantly of *Sattva, Rajas*, or *Tamas* type, depending on their total score on the test. The relationship between the *guna* type and the score is summarized below:

**Table:** 

Score	< 24	24–28	> 28
*Guna* Type	*Tamas*	*Rajas*	*Sattva*

SEQ: This multidimensional self esteem questionnaire has 83 items with seven subscales in the form of statements with both positive and negative items. Thirty-two items have negative scoring. Assessment is done on a 4-point scale to evaluate competency (COMP), global self esteem (GSE), moral and self control (MSE), social esteem (SSE), family scale (FSE), body and physical appearance (BPA), and the lie scale (LIS). A higher score indicates greater predominance of that mode.

#### Data analysis

Data was analyzed using SPSS version 10.0.

A sample size of 164 was calculated based on previous studies[22] that showed an effect size of 0.8 with a power of 0.8 and alpha set to 0.05. The minimum sample size was found to be 164. This calculation was done using G power.[23] The size of the sample that was actually used was 174.

Baseline data for both the groups were assessed for normal distribution using the Kolmogorov Smirnov test. Independent samples’ t-test was done for checking the homogeneity of baseline scores of the two groups.

The GIN data was analyzed using McNemar’s test. As the baseline values of SEQ were not normally distributed, the Wilcoxon signed ranks’ test was used to compare means within the group and the Mann Whitney U test to compare the means between the groups. As the study population had a wide age range, statistical analysis was also carried out by grouping them as juniors (age ≤ 24 years) and seniors (age > 24 years). The independent samples’ t-test for between groups’ and Paired Samples Test for within groups’ comparisons was conducted for the two age groups. The data was also analyzed using gender as a factor.

## RESULTS

**Figure 1:**
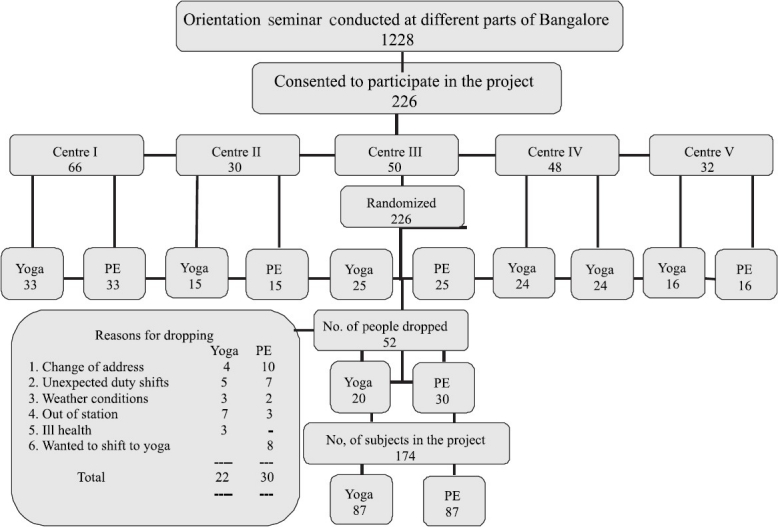
The study profile

Figure 1 shows the study profile. Of the 1228 subjects who attended the motivational lectures, 226 who satisfied the inclusion and exclusion criteria were selected and randomly allotted to Y and PE groups. The reasons for dropout of 52 subjects are shown in Figure 1. The analysis was carried out on data on 174 (Y = 87 and PE = 87) subjects.

Table 2 shows the demographic data. There were 87 females and males within the age range of 18–71 years; the mean age was 29.47 ± 12.20 years. They belonged to different callings: some were college and training college students, others were engaged in different professions and occupations, while still others were enjoying retirement.

**Table 2:** Demographic data for the 174 subjects

	Yoga (*n* = 87)	PE (*n* = 87)
Age (years)	28.72 ± 12.24	30.49 ± 12.68
Range	18–71 yrs	18–66 yrs
Female	39	38
Male	48	49
Categories		
Students	49	44
Employees	18	30
Housewives	10	7
Business	10	6

### Gita inventory of personality

The Gita inventory of personality GIN gives a single overall score that describes the disposition of an individual towards *Sattva, Rajas*, or *Tamas*. The ranges are 29–30 for *Sattva*, 24–28 for Rajas, and Tamas for scores < 24. Table 3 describes the number of people who changed over from one *guna* type to another after the interventions, based on this characterization.

**Table 3:** Number of persons in three guna domains before and after the intervention in the two groups

Personality	Yoga		PE	
	Before	After	Before	After
*Sattva*	8	17	5	9
*Rajas*	54	57	60	62
*Tamas*	25	13	22	16
Total	87	87	87	87

The question of interest was whether an individual’s *guna* type changes after intervention. To ascertain this, three different McNemar’s tests were done to check the change from the presence of a *guna* to its absence. Thus, the McNemar’s test was done to ascertain the significance of shifts of the number of subjects who were predominantly of non*Sattva* type before intervention, to the *Sattva* type after intervention. The same was done for non*Rajas* to *Rajas*, and from non *Tamas* to *Tamas* types.

A cross-tabulation of the shifts of the *guna* types for each group is presented in Table 4. The cross-tabulation [Table 4] is to be interpreted as follows: in the yoga group, three subjects who were of the *Sattva* type remained so after intervention. Sixty-five who were non*Sattva types remained* so. Fourteen subjects who were non*Sattva* types before, became *Sattva* types afterwards, whereas five who were Sattva types before, became non*Sattva* types after intervention. A similar interpretation holds for *Rajas* and *Tamas* data.

**Table 4:** Cross-tabulation of *Sattva, Rajas,* and *Tamas* before and after in the Yoga group

	Sattva after	Non Sattva after		Rajas after	Non Rajas after		Tamas after	Non Tamas after
Sattva before	3	5	Rajas before	35	19	Tamas before	7	18
Non Sattva before	14	65	Non Rajas before	22	11	Non Tamas before	6	56

### Self esteem questionnaire

The results of the Self Esteem Questionnaire (SEQ) administered to the subjects are summarized below:

**Competency (COM)**: The PE group (scores of 43.44–45.23) (*P* = 0.033) and juniors in the PE group (scores of 42.34–45.07) (*P* = 0.035) showed a significant increase in competency. The yoga group also showed an increase but it was not significant.

**Global self esteem (GSE)**: The Y group (scores of 46.68–49.47) (*P* = 0.036) showed a significant increase in global self esteem. Seniors (45.53–51.25) (*P* = 0.030) and females (45.00 to 50.49) in the yoga group also showed significant increases. The PE group also showed an increase in global self esteem but it was not significant.

**Moral and self esteem (MSE)**: Significant changes occurred in the yoga group (scores of 34.78–37.31) (*P* = 0.003), seniors (34.31–39.39) (*P* = 0.004) and females (33.59–37.77) (*P* = 0.001). The PE group also showed an increase in moral and self esteem but it was not significant.

**Social esteem (SSE)**: Significant changes occurred in seniors (31.39–33.92) (*P* = 0.023) and females (31.28–33.41) (*P* = 0.006) in the yoga group. The PE group also showed an increase in social esteem but it was not significant.

**Family self esteem (FSE)**: A significant increase was noticed in seniors (34.83–38.11) (*P* = 0.002) and females (35.15–37.36) (*P* = 0.003) in the yoga group. The PE group also showed an increase in family self esteem but it was not significant.

**Body and physical appearance (BPA)**: A significant improvement was noticed in the Yoga group (24.56–26.23) (*P* = 0.003). Juniors (25.93–26.59) (*P* = 0.002) and males (26.37–25.90) (*P* = 0.047) in the PE group showed a significant increase.

### Correlations

No significant correlations were found between any of the measures of the Self Esteem questionnaire and GIN according to the Pearson Correlation test.

## DISCUSSION

This was a randomized, controlled, prospective study in normal adults comparing the efficacy of yoga with a control group practicing physical exercises. This study has demonstrated that an eight weeks-long intervention of an integrated yoga module improved and self esteem.

McNemar’s test, a nonparametric test, is used to determine if the changes of yoga group (predominance of a *guna*, or its absence) noticed are significant. Table 5 shows that there is a significant reduction in the manifestation of the *Tamas* type (*P* = 0.023) [Table 6] and a marginally significant increase in the manifestation of the *Sattva* type (*P* = 0.064) in the Yoga group. The *Rajas* type people however, did not change significantly. In the PE group, no significant changes are seen for any of the transition [Table 7]. Thus, in the Y group, there were a significant number (14) of subjects who shifted from non*Sattva* to *Sattva* type, and from *Tamas* to non *Tamas* (18). The upward trend in the central tendency of the scores on the GIN seems to be quite consistent with the Gita concept. This concept proposes that the *Gunas* initially vary in their dominance in determining the personality of an individual (canto 14),[16] but that gradually the individual’s personality mostly settles on one of the *Gunas* (canto 14)[16] and ultimately, though very slowly, through a sort of moral evolution, moves from *Tamas* and *Rajas* to *Sattva*, and finally goes beyond the *Gunas* and attains liberation (cantos 7 and 14).[17] This trend of shift towards an increase in *Sattva* and a decrease in *Tamas* after eight weeks of integrated yoga practices, but not after PE, is clearly demonstrated in this study.

Furthermore, in the famous scriptural text, the *Gita* a *guna* indicates a specific behavioral style. *Sattva* indicates purity, wisdom, bliss, serenity, love of knowledge, spiritual excellence, and such other noble and sublime qualities. *Rajas* indicates egoism, activity, restlessness, and hankering after mundane things such as wealth, power, valor, and comforts. *Tamas* implies qualities such as bias, heedlessness, and inertia, besides perversion in taste, thought and action.[14] *Rajas* and *Tamas* are said to be the manifestations of a violent state of mind in which a person lacks mastery over upsurges of emotions and impulsive behavior. Yoga is known to be useful in promoting positive health at the physical, mental, social, emotional, and spiritual domains, which results in becoming energetic, confident, masterful over the sense organs, possessing harmony and coordination between right and left brain functions, as well as becoming stress-free. These are attributed to the calmness of the mind leading to forbearance and stability of the nervous system,[24] which are the qualities of a *Sattva* -dominant person.

A similar study by Dasa[25] was conducted by the use of *mahamantra* (chanting of a specific pious chant) and the Vedic Personality Inventory in a three-armed, randomized, prospective, controlled study on 62 volunteers. They showed that the *mahamantra* group had increased *Sattva* and decreased *Tamas* with no significant change in *Rajas* scores, which is also a measure of the three *Gunas*. In their study, they also showed a significant reduction in stress, anxiety, and depression after a month of chanting *mahamantra*, 20 minutes daily for four weeks. The present study has also demonstrated a significant improvement in *Sattva* and *Tamas* scores and a reduction in *Rajas* in females and seniors, which was not found in the earlier study conducted by Dasa. This difference would be because of the inclusion of *Asanas* and *Pranayama* in addition to the meditation technique in the present study, whereas the *mahamantra* study was mainly a form of meditation.

In the self esteem components, the Yoga group showed significant increases in GSE, MSE, and BPA whereas the physical exercises group showed significant increases in only one subscale of self esteem—COM [Table 8]. Although there is a statistically significant difference in the mean scores of these parameters before and after intervention for both groups, it is the measure of the effect size that is relevant. The concept of effect size in populations for a parameter is a concept that was first introduced by the sociologist, Cohen.[26] The effect size is an absolute measure of the difference that exists between populations for a parameter. Table 7 shows that the effect sizes are clearly larger for the group that practised yoga.

**Table 5:** Cross-tabulation of *Sattva, Rajas*, and *Tamas* before and after in the PE group

	Sattva after	Non Sattva after		Rajas after	Non Rajas after		Tamas after	Non Tamas after
Sattva before	4	1	Rajas before	48	12	Tamas before	9	13
Non Sattva before	5	77	Non Rajas Before	14	13	Non Tamas before	7	58

**Table 6:** Results of McNemar's test

		Sattva—	Rajas—	Tamas—
		Non Sattva	Non Rajas	Non Tamas
	No.	87	87	87
Significance	Y	0.064	0.755	0.023
	PE	0.219	0.845	0.263

**Table 7:** Effect size for Self esteem domains

BPA		GSE		MSC	
Yoga	PE	Yoga	PE	Yoga	PE
0.33	0.04	0.21	0.16	0.32	0.01

(Effect size = difference in means (after – before)/SD of the difference scores), GSE - Global self esteem, BPA - Body and physical appearance, MSC - Moral and self esteem

**Table 8:** Descriptive statistics for self esteem

	Yoga			PE		
	Mean ± SD before	Mean ± SD after	*P* value	Mean ± SD before	Mean ± SD after	*P* value
COM	42.90 ± 6.90	44.47 ± 6.66	0.105	43.44 ± 7.04	45.23 ± 6.08	0.033
GSE	46.68 ± 10.43	49.47 ± 8.41	0.036	48.67 ± 8.51	50.39 ± 9.13	0.129
MSE	34.78 ± 6.10	37.31 ± 5.79	0.003	37.51 ± 5.03	37.40 ± 6.36	0.762
BPA	24.56 ± 5.15	26.23 ± 4.29	0.003	25.55 ± 5.49	25.79 ± 4.61	0.705

COM - Competency, GSE - Global self esteem, MSE - Moral and self esteem, BPA - Body and physical appearance

An analysis based on gender showed significant changes in Y group females for GSE, MSE, SSE, and FSE and for PE group females for COM. In males, significant changes were observed in BPA in the PE Group. Age analysis showed differences within the two age groups (≤ 24 and > 24 years). The older group showed better changes than the younger group within the Yoga group. In the younger age group, most significant changes observed in the PE group were for COM and BPA. In the older group, significant changes observed in the Yoga group were for GSE, MSE, SSE, and FSE [Table 9].

**Table 9:** Descriptive statistics for self esteem: Age and gender distributions

Domains		Yoga			PE		
		Mean ± SD before	Mean ± SD after	*P* value	Mean ± SD before	Mean ± SD after	*P* value
Age ≤ 24 years	COM	42.86 ± 6.14	43.67 ± 6.71	0.407	42.34 ± 7.82	45.07 ± 6.47	0.035
	BPA	24.98 ± 5.54	26.20 ± 4.62	0.145	25.93 ± 6.58	26.59 ± 5.11	0.002
Age > 24 years	GSE	45.53 ± 12.19	51.25 ± 8.46	0.030	49.93 ± 9.28	51.09 ± 8.63	0.364
	MSE	34.31 ± 6.92	39.39 ± 5.59	0.004	38.19 ± 5.23	38.33 ± 5.55	0.805
	SSE	31.39 ± 5.48	33.92 ± 4.23	0.023	33.90 ± 4.77	34.09 ± 4.51	0.499
	FSE	34.83 ± 10.38	38.11 ± 8.69	0.002	36.86 ± 6.46	37.98 ± 6.78	0.905
Females	GSE	45.00 ± 12.04	50.49 ± 8.51	0.045	48.68 ± 9.35	51.32 ± 9.01	0.125
	MSE	33.59 ± 7.10	37.77 ± 6.46	0.001	37.08 ± 5.49	38.42 ± 5.46	0.171
	SSE	31.28 ± 5.80	33.41 ± 4.38	0.006	32.63 ± 4.56	34.03 ± 4.86	0.156
	FSE	35.15 ± 9.67	37.36 ± 8.87	0.003	37.26 ± 6.37	37.26 ± 7.63	0.259
Males	BPA	25.79 ± 4.07	26.77 ± 3.79	0.382	26.37 ± 6.10	25.90 ± 5.05	0.047

COM - Competency, GSE - Global self esteem, MSE - Moral and self esteem, BPA - Body and physical appearance, SSE - Social esteem, FSE - Family self esteem

Relatively better changes in the Y group (compared to PE) can be noted in the GSC (general appraisal of the self based on the evolution of all parts of one’s self) and MSC (which is the reflection of feeling good, as in being honest, sincere, adhering to social values etc.) domains [Table 7]. The practice of breathing exercises, *pranayama*, and the systematic breathing during *asanas* regularizes the breathing mechanism, trains the proper use of abdominal and chest muscles, and also improves the vital capacity and stamina, which in turn can influence better self esteem.[27] Yogic breathing exercises positively affect the mood and they have clinical potential as self-control techniques for improving and stabilizing affective states.[28]

COM (the ability to evaluate and understand one’s personal resources), BPA (body image as a contribution of physical appearance and capabilities) in younger groups and BPA in males were significantly improved in the PE group. Atlantis *et al*. studied the efficacy of exercise-based intervention of eight weeks’ duration in a population of Australian employees and showed that the intervention significantly improved the QoL in comparison to a waiting list control group (measured by SF-20). They showed an improvement of 12.8% in physical functioning, 9.90% in general health, 44.50% in vitality, and 15.90% in mental health scores.[22]

Thus, Yoga helps in the improvement in *Gunas* (personality) and self esteem. These findings reveal that Yoga has greater influence on holistic personality growth (*Gunas*) when compared to routine physical exercise. Hence, it can be considered independently to promote quality of life and health, prevent chronic diseases, and to promote socio-economic development.

Finally, the fact that there were no significant correlations between the Gita Inventory and the parameters of the Self Esteem questionnaire is noteworthy. This lack of correlation between the two instruments shows that they measure orthogonal qualities.

Furthermore, we can observe that, like physical exercise, Yoga is traditionally acceptable in India, is easy and cost-effective to perform, and has become popular around the globe. Yet another point of this design is a matched intervention in the form of exercises. However, a third group with no intervention would have given greater insights to the comparisons.

In summary, this randomized interventional study has shown that the improvement in the Yoga group is more when compared to the physical exercises’ group for all the *Gunas*, with accompanying promotion of positive health and self esteem. This study thus provides scientific evidence to consider yoga as an independent practice that can be beneficial in improving one’s quality of life.
